# 1-(3,4-Di­meth­oxy­phen­yl)-3-phenyl­prop-2-en-1-one

**DOI:** 10.1107/S160053681400378X

**Published:** 2014-02-28

**Authors:** Basavaiah Umesha, Yeriyur Basavaiah Basavaraju, Manpreet Kaur, Hemmige S. Yathirajan, Jerry P. Jasinski

**Affiliations:** a Department of Studies in Chemistry, University of Mysore, Manasagangotri, Mysore 570 006, India; b Department of Chemistry, Keene State College, 229 Main Street, Keene, NH 03435-2001, USA

## Abstract

In the title compound, C_17_H_16_O_3_, the dihedral angle between the mean planes of the benzene rings is 57.1 (1)°. The mean plane of the ketone group is twisted by 10.0 (5)° from that of the di­meth­oxy­phenyl ring. The two di­meth­oxy­phenyl groups are twisted slighly from the mean plane of the phenyl ring, with C—O—C—C torsion angles of 6.4 (2) and −7.9 (2)° [r.m.s. deviations = 0.15 (3) and 0.18 (3) Å for the two methoxy C atoms]. In the crystal, weak centroid–centroid π–π stacking inter­actions, with inter­centroid distances of 3.8939 (11) and 3.9430 (10) Å are observed.

## Related literature   

For applications of the title compound as an inter­mediate in the synthesis of pyrazole derivatives, which have pharmaceutical applications, see: Basavaraju & Devaraju (2002 ▶). For applications of chalcone derivatives in biological studies, see: Choudhary & Juyal (2011 ▶). For applications of chalcone derivatives for their blue-light transmittance, see: Uchida *et al.* (1998 ▶). For the synthesis, see: Umesha & Basavaraju (2013 ▶). For standard bond lengths, see: Allen *et al.* (1987 ▶).
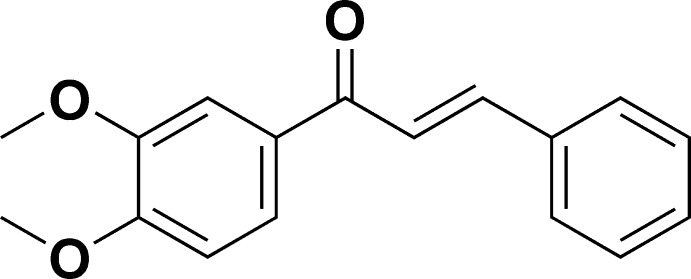


## Experimental   

### 

#### Crystal data  

C_17_H_16_O_3_
*M* *_r_* = 268.30
Triclinic, 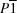

*a* = 7.3269 (7) Å
*b* = 9.8923 (11) Å
*c* = 10.7668 (11) Å
α = 102.408 (9)°
β = 109.255 (9)°
γ = 98.951 (9)°
*V* = 697.48 (13) Å^3^
*Z* = 2
Cu *K*α radiation
μ = 0.70 mm^−1^
*T* = 173 K
0.36 × 0.28 × 0.16 mm

#### Data collection  

Agilent Xcalibur (Eos, Gemini) diffractometer
Absorption correction: multi-scan (*CrysAlis PRO* and *CrysAlis RED*; Agilent, 2012 ▶) *T*_min_ = 0.842, *T*_max_ = 1.000
4094 measured reflections
2671 independent reflections
2261 reflections with *I* > 2σ(*I*)
*R*_int_ = 0.034

#### Refinement  

*R*[*F* ^2^ > 2σ(*F* ^2^)] = 0.051
*wR*(*F* ^2^) = 0.152
*S* = 1.05
2671 reflections
184 parameters
H-atom parameters constrained
Δρ_max_ = 0.30 e Å^−3^
Δρ_min_ = −0.20 e Å^−3^

### 

Data collection: *CrysAlis PRO* (Agilent, 2012 ▶); cell refinement: *CrysAlis PRO*; data reduction: *CrysAlis RED* (Agilent, 2012 ▶); program(s) used to solve structure: *SUPERFLIP* (Palatinus & Chapuis, 2007 ▶; Palatinus & van der Lee, 2008 ▶; Palatinus *et al.*, 2012 ▶); program(s) used to refine structure: *SHELXL97* (Sheldrick, 2008 ▶); molecular graphics: *OLEX2* (Dolomanov *et al.*, 2009 ▶); software used to prepare material for publication: *OLEX2*.

## Supplementary Material

Crystal structure: contains datablock(s) I. DOI: 10.1107/S160053681400378X/bt6962sup1.cif

Structure factors: contains datablock(s) I. DOI: 10.1107/S160053681400378X/bt6962Isup2.hkl

Supporting information file. DOI: 10.1107/S160053681400378X/bt6962Isup3.cml

CCDC reference: 987727

Additional supporting information:  crystallographic information; 3D view; checkCIF report

## Figures and Tables

**Fig. 1.**
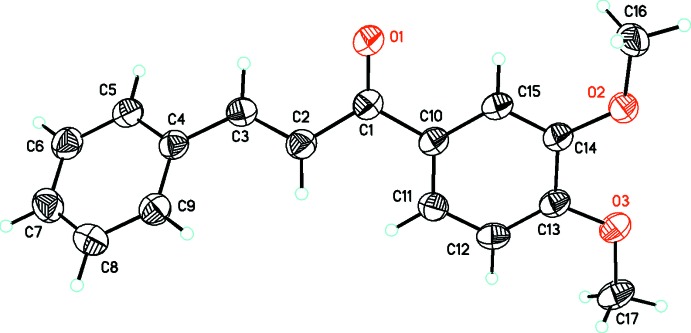
ORTEP drawing of (I), C17H16O3, showing the labeling scheme with 50% probability displacement ellipsoids.

**Fig. 2.**
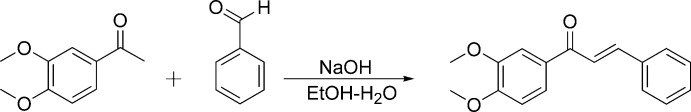
The formation of the title compound.

## supplementary crystallographic information

### 1. Comment

1-(3,4-dimethoxyphenyl)-3-phenylprop-2-en-1-one is an intermediate for the synthesis of pyrazole derivatives, podophyllotoxin and its derivatives which have pharmaceutical applications (Basavaraju & Devaraju, 2002). More generally, chalcone derivatives have many applications in biological studies (Choudhary & Juyal, 2011). Chalcone derivatives are notable for their excellent blue-light transmittance and good crystallizability (Uchida *et al.*, 1998). In continuation of our work on chalcone derivatives, this paper reports the crystal structure of the title compound C_17_H_16_O_3_.

In the title compound the dihedral angle between the mean planes of the two phenyl rings is 57.1 (1)° (Fig. 1). The mean plane of the ketone group (C2/C1/O1/C10) is twisted by 10.0 (5)° from that of the dimethoxyphenyl ring. The two dimethoxyphenyl groups are twisted slighly from the mean plane of the phenyl ring with torsion angles of 6.4 (2)° (C16/O2/C14/C15) and -7.9 (2)° (C17/O3/C13/C12) [r.m.s. deviations = 0.15 (3) and 0.18 (3)Å for the two methoxy C atoms]. Bond lengths are in normal ranges (Allen *et al.*, 1987). In the crystal, while no classical hydrogen bonds are observed, weak *Cg*1–*Cg*1 and *Cg*2–*Cg*2 π–π stacking interactions with intercentroid distances of 3.8939 (11) Å and 3.9430 (10) Å (Symmetry operations 1 - *x*, 2 - *y*, 1 - *z* and -*x*, 1 - *y*, 2 - *z*; *Cg*1 and *Cg*2 are the centroids of the phenyl rings, C4–C9 and C10–C15) between the phenyl rings are observed and contribute to the crystal packing.

### 2. Experimental

1-(3,4-Dimethoxyphenyl)ethanone (1.80 g 10 mmol) and benzaldehyde (1.02 ml, 10 mmol) were stirred in water (40 ml) and ethanol (20 ml) mixture in the presence of sodium hydroxide (0.8 g, 20 mmol) at 288–303 K for 4 h (Fig. 2). The reaction mixture was kept overnight in an ice bath. The precipitated products were filtered and recrystallized from methanol. Anal. calcd. for C_17_H_16_O_3_: C, 76.10; H, 6.01. Found: C, 76.04; H, 5.89% (Umesha & Basavaraju, 2013).

### 3. Refinement

All of the H atoms were placed in their calculated positions and then refined using the riding model with C—H lengths of 0.95 Å (CH) or 0.98 Å (CH_3_). Isotropic displacement parameters for these atoms were set to 1.2 (CH) or 1.5 (CH_3_) times *U*_eq_ of the parent atom. Idealized Me were refined as a rotating group.

### Crystal data

**Table:**

C_17_H_16_O_3_	*Z* = 2
*M**_r_* = 268.30	*F*(000) = 284
Triclinic, *P*1	*D*_x_ = 1.278 Mg m^−^^3^
*a* = 7.3269 (7) Å	Cu *K*α radiation, λ = 1.54184 Å
*b* = 9.8923 (11) Å	Cell parameters from 1960 reflections
*c* = 10.7668 (11) Å	θ = 4.5–72.3°
α = 102.408 (9)°	µ = 0.70 mm^−^^1^
β = 109.255 (9)°	*T* = 173 K
γ = 98.951 (9)°	Irregular, orange
*V* = 697.48 (13) Å^3^	0.36 × 0.28 × 0.16 mm

### Data collection

**Table:**

Agilent Xcalibur (Eos, Gemini) diffractometer	2671 independent reflections
Radiation source: Enhance (Cu) X-ray Source	2261 reflections with *I* > 2σ(*I*)
Graphite monochromator	*R*_int_ = 0.034
Detector resolution: 16.0416 pixels mm^-1^	θ_max_ = 72.4°, θ_min_ = 4.5°
ω scans	*h* = −8→8
Absorption correction: multi-scan (*CrysAlis PRO* and *CrysAlis RED*; Agilent, 2012)	*k* = −11→12
*T*_min_ = 0.842, *T*_max_ = 1.000	*l* = −12→13
4094 measured reflections	

### Refinement

**Table:**

Refinement on *F*^2^	Hydrogen site location: inferred from neighbouring sites
Least-squares matrix: full	H-atom parameters constrained
*R*[*F*^2^ > 2σ(*F*^2^)] = 0.051	*w* = 1/[σ^2^(*F*_o_^2^) + (0.0909*P*)^2^ + 0.0371*P*] where *P* = (*F*_o_^2^ + 2*F*_c_^2^)/3
*wR*(*F*^2^) = 0.152	(Δ/σ)_max_ < 0.001
*S* = 1.05	Δρ_max_ = 0.30 e Å^−^^3^
2671 reflections	Δρ_min_ = −0.20 e Å^−^^3^
184 parameters	Extinction correction: *SHELXL97* (Sheldrick, 2008), Fc^*^=kFc[1+0.001xFc^2^λ^3^/sin(2θ)]^-1/4^
0 restraints	Extinction coefficient: 0.016 (3)
Primary atom site location: structure-invariant direct methods	

### Special details

**Table:**

Geometry. All e.s.d.'s (except the e.s.d. in the dihedral angle between two l.s. planes) are estimated using the full covariance matrix. The cell e.s.d.'s are taken into account individually in the estimation of e.s.d.'s in distances, angles and torsion angles; correlations between e.s.d.'s in cell parameters are only used when they are defined by crystal symmetry. An approximate (isotropic) treatment of cell e.s.d.'s is used for estimating e.s.d.'s involving l.s. planes.

### Fractional atomic coordinates and isotropic or equivalent isotropic displacement parameters (Å^2^)

**Table:**

	*x*	*y*	*z*	*U*_iso_*/*U*_eq_	
O1	0.1915 (2)	0.40453 (12)	0.63226 (11)	0.0458 (4)	
O2	0.11843 (19)	0.13233 (12)	0.97010 (11)	0.0408 (3)	
O3	0.17608 (19)	0.32198 (13)	1.19243 (11)	0.0412 (3)	
C1	0.2507 (2)	0.49627 (17)	0.74331 (15)	0.0340 (4)	
C2	0.3317 (2)	0.64736 (17)	0.75594 (16)	0.0354 (4)	
H2	0.4221	0.7092	0.8425	0.042*	
C3	0.2797 (2)	0.69778 (17)	0.64760 (15)	0.0349 (4)	
H3	0.1903	0.6322	0.5627	0.042*	
C4	0.3472 (2)	0.84500 (16)	0.64690 (15)	0.0335 (4)	
C5	0.2255 (3)	0.89601 (17)	0.54684 (16)	0.0379 (4)	
H5	0.1058	0.8340	0.4781	0.046*	
C6	0.2791 (3)	1.03693 (18)	0.54759 (17)	0.0426 (4)	
H6	0.1942	1.0718	0.4806	0.051*	
C7	0.4548 (3)	1.12685 (18)	0.64490 (18)	0.0439 (4)	
H7	0.4906	1.2234	0.6450	0.053*	
C8	0.5793 (3)	1.07642 (18)	0.74269 (17)	0.0423 (4)	
H8	0.7013	1.1381	0.8089	0.051*	
C9	0.5262 (2)	0.93635 (17)	0.74404 (16)	0.0375 (4)	
H9	0.6118	0.9022	0.8113	0.045*	
C10	0.2372 (2)	0.45707 (16)	0.86635 (15)	0.0315 (4)	
C11	0.2692 (2)	0.55923 (17)	0.98761 (16)	0.0349 (4)	
H11	0.3050	0.6579	0.9945	0.042*	
C12	0.2489 (2)	0.51738 (17)	1.09853 (15)	0.0361 (4)	
H12	0.2698	0.5877	1.1807	0.043*	
C13	0.1986 (2)	0.37465 (17)	1.09029 (15)	0.0335 (4)	
C14	0.1668 (2)	0.26993 (17)	0.96737 (15)	0.0322 (4)	
C15	0.1855 (2)	0.31230 (16)	0.85772 (14)	0.0318 (4)	
H15	0.1630	0.2423	0.7750	0.038*	
C16	0.1048 (3)	0.02320 (18)	0.85413 (17)	0.0456 (5)	
H16A	−0.0049	0.0246	0.7726	0.068*	
H16B	0.0797	−0.0701	0.8711	0.068*	
H16C	0.2303	0.0402	0.8391	0.068*	
C17	0.2323 (3)	0.4222 (2)	1.32445 (16)	0.0475 (5)	
H17A	0.1481	0.4902	1.3175	0.071*	
H17B	0.3723	0.4738	1.3562	0.071*	
H17C	0.2151	0.3711	1.3899	0.071*	

### Atomic displacement parameters (Å^2^)

**Table:**

	*U*^11^	*U*^22^	*U*^33^	*U*^12^	*U*^13^	*U*^23^
O1	0.0689 (9)	0.0374 (6)	0.0314 (6)	0.0090 (6)	0.0214 (6)	0.0095 (5)
O2	0.0594 (8)	0.0336 (6)	0.0330 (6)	0.0083 (5)	0.0237 (5)	0.0092 (5)
O3	0.0523 (7)	0.0461 (7)	0.0281 (6)	0.0104 (5)	0.0194 (5)	0.0106 (5)
C1	0.0356 (8)	0.0368 (8)	0.0314 (8)	0.0102 (6)	0.0135 (6)	0.0106 (6)
C2	0.0359 (8)	0.0376 (9)	0.0319 (8)	0.0070 (6)	0.0134 (6)	0.0092 (6)
C3	0.0385 (8)	0.0343 (8)	0.0316 (7)	0.0085 (6)	0.0145 (6)	0.0068 (6)
C4	0.0388 (8)	0.0342 (8)	0.0310 (7)	0.0108 (6)	0.0173 (6)	0.0085 (6)
C5	0.0416 (9)	0.0390 (9)	0.0313 (8)	0.0092 (7)	0.0124 (6)	0.0088 (6)
C6	0.0504 (10)	0.0417 (9)	0.0383 (9)	0.0158 (7)	0.0152 (7)	0.0156 (7)
C7	0.0540 (10)	0.0326 (8)	0.0486 (9)	0.0113 (7)	0.0233 (8)	0.0116 (7)
C8	0.0388 (9)	0.0385 (9)	0.0432 (9)	0.0048 (7)	0.0130 (7)	0.0060 (7)
C9	0.0377 (9)	0.0390 (9)	0.0362 (8)	0.0109 (7)	0.0133 (7)	0.0117 (6)
C10	0.0305 (7)	0.0362 (8)	0.0285 (7)	0.0090 (6)	0.0117 (6)	0.0090 (6)
C11	0.0351 (8)	0.0332 (8)	0.0340 (8)	0.0060 (6)	0.0128 (6)	0.0068 (6)
C12	0.0373 (8)	0.0386 (9)	0.0287 (7)	0.0085 (7)	0.0131 (6)	0.0017 (6)
C13	0.0330 (8)	0.0409 (8)	0.0271 (7)	0.0084 (6)	0.0132 (6)	0.0085 (6)
C14	0.0317 (8)	0.0356 (8)	0.0288 (7)	0.0077 (6)	0.0116 (6)	0.0084 (6)
C15	0.0325 (8)	0.0362 (8)	0.0254 (7)	0.0081 (6)	0.0111 (6)	0.0060 (6)
C16	0.0664 (12)	0.0332 (8)	0.0426 (9)	0.0097 (8)	0.0303 (9)	0.0079 (7)
C17	0.0549 (11)	0.0619 (11)	0.0258 (8)	0.0141 (9)	0.0185 (7)	0.0078 (7)

### Geometric parameters (Å, º)

**Table:**

O1—C1	1.2290 (18)	C8—H8	0.9500
O2—C14	1.3613 (19)	C8—C9	1.384 (2)
O2—C16	1.4282 (18)	C9—H9	0.9500
O3—C13	1.3592 (18)	C10—C11	1.392 (2)
O3—C17	1.4331 (19)	C10—C15	1.398 (2)
C1—C2	1.479 (2)	C11—H11	0.9500
C1—C10	1.487 (2)	C11—C12	1.389 (2)
C2—H2	0.9500	C12—H12	0.9500
C2—C3	1.331 (2)	C12—C13	1.378 (2)
C3—H3	0.9500	C13—C14	1.418 (2)
C3—C4	1.466 (2)	C14—C15	1.375 (2)
C4—C5	1.396 (2)	C15—H15	0.9500
C4—C9	1.397 (2)	C16—H16A	0.9800
C5—H5	0.9500	C16—H16B	0.9800
C5—C6	1.386 (2)	C16—H16C	0.9800
C6—H6	0.9500	C17—H17A	0.9800
C6—C7	1.378 (3)	C17—H17B	0.9800
C7—H7	0.9500	C17—H17C	0.9800
C7—C8	1.387 (2)		
			
C14—O2—C16	117.05 (12)	C11—C10—C15	119.33 (14)
C13—O3—C17	117.20 (13)	C15—C10—C1	118.37 (13)
O1—C1—C2	120.69 (14)	C10—C11—H11	119.9
O1—C1—C10	120.23 (14)	C12—C11—C10	120.20 (15)
C2—C1—C10	119.06 (13)	C12—C11—H11	119.9
C1—C2—H2	119.6	C11—C12—H12	119.7
C3—C2—C1	120.82 (14)	C13—C12—C11	120.51 (14)
C3—C2—H2	119.6	C13—C12—H12	119.7
C2—C3—H3	116.9	O3—C13—C12	125.41 (14)
C2—C3—C4	126.11 (14)	O3—C13—C14	114.96 (14)
C4—C3—H3	116.9	C12—C13—C14	119.63 (14)
C5—C4—C3	118.50 (14)	O2—C14—C13	114.95 (13)
C5—C4—C9	119.08 (15)	O2—C14—C15	125.58 (14)
C9—C4—C3	122.41 (14)	C15—C14—C13	119.47 (14)
C4—C5—H5	119.9	C10—C15—H15	119.6
C6—C5—C4	120.12 (15)	C14—C15—C10	120.86 (14)
C6—C5—H5	119.9	C14—C15—H15	119.6
C5—C6—H6	119.8	O2—C16—H16A	109.5
C7—C6—C5	120.37 (16)	O2—C16—H16B	109.5
C7—C6—H6	119.8	O2—C16—H16C	109.5
C6—C7—H7	120.0	H16A—C16—H16B	109.5
C6—C7—C8	120.02 (16)	H16A—C16—H16C	109.5
C8—C7—H7	120.0	H16B—C16—H16C	109.5
C7—C8—H8	119.9	O3—C17—H17A	109.5
C9—C8—C7	120.13 (16)	O3—C17—H17B	109.5
C9—C8—H8	119.9	O3—C17—H17C	109.5
C4—C9—H9	119.9	H17A—C17—H17B	109.5
C8—C9—C4	120.23 (15)	H17A—C17—H17C	109.5
C8—C9—H9	119.9	H17B—C17—H17C	109.5
C11—C10—C1	122.28 (14)		
			
O1—C1—C2—C3	24.6 (2)	C5—C6—C7—C8	0.1 (3)
O1—C1—C10—C11	−168.54 (15)	C6—C7—C8—C9	−0.9 (3)
O1—C1—C10—C15	9.7 (2)	C7—C8—C9—C4	0.1 (3)
O2—C14—C15—C10	−179.58 (14)	C9—C4—C5—C6	−2.3 (2)
O3—C13—C14—O2	0.0 (2)	C10—C1—C2—C3	−153.98 (15)
O3—C13—C14—C15	179.85 (13)	C10—C11—C12—C13	0.5 (2)
C1—C2—C3—C4	179.22 (14)	C11—C10—C15—C14	−0.3 (2)
C1—C10—C11—C12	178.00 (14)	C11—C12—C13—O3	179.57 (15)
C1—C10—C15—C14	−178.63 (13)	C11—C12—C13—C14	−0.2 (2)
C2—C1—C10—C11	10.0 (2)	C12—C13—C14—O2	179.81 (14)
C2—C1—C10—C15	−171.68 (13)	C12—C13—C14—C15	−0.4 (2)
C2—C3—C4—C5	−154.28 (17)	C13—C14—C15—C10	0.6 (2)
C2—C3—C4—C9	24.4 (2)	C15—C10—C11—C12	−0.3 (2)
C3—C4—C5—C6	176.43 (15)	C16—O2—C14—C13	−173.81 (14)
C3—C4—C9—C8	−177.18 (15)	C16—O2—C14—C15	6.4 (2)
C4—C5—C6—C7	1.5 (3)	C17—O3—C13—C12	−7.9 (2)
C5—C4—C9—C8	1.5 (2)	C17—O3—C13—C14	171.84 (14)
